# Freshwater habitats within the Natura 2000 network

**DOI:** 10.1002/eap.70241

**Published:** 2026-04-22

**Authors:** Annette Baattrup‐Pedersen, Marta Baumane, Anders Nielsen, Dennis Trolle, Paulo Branco, Florian Borgwardt, Daniel Hering, Sebastian Birk

**Affiliations:** ^1^ Department of Biology Aarhus University Aarhus C Denmark; ^2^ WateriTech Skanderborg Denmark; ^3^ Forest Research Centre, Associate Laboratory TERRA School of Agriculture, University of Lisbon Lisbon Portugal; ^4^ University of Natural Resources and Life Sciences, Vienna Vienna Austria; ^5^ Faculty of Biology, Aquatic Ecology University of Duisburg‐Essen Essen Germany

**Keywords:** biodiversity, conservation, freshwater habitats, Habitats Directive, Nature Restoration Law

## Abstract

Freshwater biodiversity is experiencing dramatic declines. Despite improvements, the trend remains negative, underlining that effective and coordinated initiatives are needed. Protected areas are considered a global cornerstone of biodiversity conservation, and in Europe, the Natura 2000 network plays a central role in safeguarding biodiversity. In the present study, we examine if designated Annex I listed freshwater habitats of the Habitats Directive are sufficiently represented to enable conservation and restoration efforts, as outlined in Article 4 of the European Nature Restoration Law (NRR), to meaningfully contribute to reversing the ongoing decline in freshwater biodiversity. Additionally, we examine whether freshwater habitats are represented across their full natural geographical range, addressing a key spatial component of long‐term persistence, as reflected in the concept of “favorable reference area” as defined in Article 3 of the NRR. We found that freshwater habitats cover 6.2% of the total area of Natura 2000 sites with running waters covering only 0.4%. In addition to a low spatial coverage, the analyses also indicated that several freshwater Annex I habitats are not represented across their full natural geographical range. Based on the obtained results, we therefore argue that a systematic strengthening of the Natura 2000 network is required, with particular emphasis on: (1) expanding the extent of designated freshwater habitat types in‐ and also outside the existing network if necessary to adequately cover freshwater habitats, and (2) identifying and addressing representation gaps across all relevant biogeographic regions. To support this process, we have developed a webtool based on the datasets underlying the analyses, which enables relevant stakeholders including EU institutions, national agencies, and local managers to examine the distribution of freshwater habitats and species protected within the current Natura 2000 network. The webtool can be accessed at https://www.waterwebtools.com/merlin.

## INTRODUCTION

Freshwater biodiversity is experiencing dramatic declines both globally and in Europe, driven by anthropogenic impacts including water abstraction, flow regulation, dam construction, pollution and widespread habitat loss (e.g., Dudgeon, 2019; Tickner et al., 2020). Moreover, freshwater biodiversity is vulnerable to anthropogenic climate change, which may have direct effects (e.g., through droughts, water temperature increase) and indirect effects by amplifying other stressors such as eutrophication (Dudgeon, 2019; Reid et al., 2019). Several EU policies work toward protection and conservation of aquatic ecosystems, and significant improvements have been made over the last decades applying various ecosystem‐based management approaches, in particular by enhancement of water quality (Haase et al., 2023; Radinger et al., 2023). Despite these improvements, the freshwater biodiversity crisis remains as 24% of decapod crustaceans, fishes and odonates on the International Union for Conservation of Nature (IUCN) Red List (RL) are threatened with extinction (Sayer et al., 2025) underlining that effective and coordinated initiatives are urgently needed (Acreman et al., 2020).

Protected areas are considered a global cornerstone of biodiversity conservation and the area of land and sea designated as formally protected has markedly increased over the past century (UNEP‐WCMC, 2024). In Europe, the Natura 2000 network has been established following the implementation of the Habitats Directive (HD) in 1992 to ensure the long‐term survival of Europe's most valuable and threatened species and habitats. The network encompasses a total of 27,000 sites covering 18% of the EU's land area and 8% of its marine territory with 230 habitat types and approximately 700 species being designated as protected due to their ecological importance, uniqueness, and role in supporting biodiversity (EC, 2025). The network plays a crucial role in the implementation of the Nature Restoration Regulation (NRR) adopted by the European Parliament in 2024. Specifically, the NRR is seeking to restore at least 30% of the EU's degraded ecosystems by 2030 and expand restoration efforts to all degraded ecosystems by 2050 (Hering et al., 2023). Furthermore, priority shall be given to restoration measures carried out in Natura 2000 sites (NRR's Article 4). The NRR is the first of its kind in setting binding targets for restoration, but the success relies not only on political commitment, stakeholder collaboration, and sufficient funding but also on identifying and implementing restoration actions in sites where the most considerable biodiversity benefits can be achieved.

Although the NRR explicitly addresses the ongoing decline in biodiversity, it cannot make a substantive contribution to halting the loss of freshwater biodiversity unless freshwater ecosystems are adequately represented within the Natura 2000 network. This prompts a critical question: to what extent can freshwater biodiversity loss be addressed through restoration actions within the existing Natura 2000 network in Europe, and is this network suitably designed to support actions that can produce meaningful outcomes for freshwater biodiversity? It has previously been argued that freshwater biodiversity is not adequately covered within the Natura 2000 network, with only 14% of European freshwater fish, 3% of nonmarine mollusks, and 19% of dragonflies listed as threatened in the IUCN RL designated under the EU HD (Acreman et al., 2020; Grantham et al., 2017; Leal et al., 2020; van Rees et al., 2021). Additionally, a study in the Iberian Peninsula highlights that the Natura 2000 network fails to adequately cover freshwater biodiversity, with some species not represented within the network and only a small fraction of species' ranges covered (Hermoso et al., 2015). Similar results have been obtained for invertebrates (Hernandez‐Manrique et al., 2012) and plants (Bagella et al., 2013). Moreover, a study from Italy revealed that the Natura 2000 network does not guarantee higher freshwater fish species richness compared to areas outside the network (Gavioli et al., 2023).

Based on the above‐mentioned studies we therefore see a need to examine the coverage and distribution of freshwater habitats within the Natura 2000 network in Europe to explore if Annex I listed habitats of the HD are sufficiently represented to enable conservation and restoration efforts, as outlined in Article 4 of the NRR, to meaningfully contribute to reversing the ongoing decline in freshwater biodiversity. Additionally, we examine whether freshwater habitats are represented across their full natural geographical range, which constitutes a key spatial element of the favorable reference area concept as defined in the NRR. While this approach does not directly assess long‐term viability, representation across the biogeographical range is commonly regarded as a supportive condition for habitat persistence, as it captures the spatial distribution of habitats within their natural range. This perspective is conceptually consistent with metapopulation theory, which highlights the importance of spatially structured habitat networks for long‐term persistence (Hanski, 1998), and relates to the favorable reference area concept without directly assessing connectivity or demographic processes. Specifically, we hypothesize that (1) protected freshwater habitats are represented with a low coverage within the Natura 2000 network (2) a majority of freshwater habitats are not represented across their full natural range within the Natura 2000 network. We use a broad definition of freshwater habitats including running and standing water habitats, peatlands, forests and coastal habitats, heathland, and grassland habitats thereby including also groundwater‐dependent terrestrial ecosystems (GWDTE), whose water requirements are protected by the Water Framework Directive (WFD; Article 1). Thereby we acknowledge that many freshwater species live in the ecotone between water and land in part of their life cycle, for example, many amphibian, insect, and fish species.

## METHODS

To describe the current status of freshwater habitats within Natura 2000 network, we extracted data from the Natura 2000 database (version 2021; EC, 2022). This database include habitat assessments for habitats included in Annex I of the HD, that is, habitats of community interest whose conservation requires the designation of special areas of conservation (SAC), for the period 2013–2018 and yearly updates from the member states, for example, on changes in habitat area. For the data analysis we created two datasets: “Freshwater, marine and coastal, and terrestrial habitats” and “Freshwater habitats” (Appendix S1: Table S2). These datasets build on “NATURA2000SITES,” “HABITATS,” and “BIOREGION” from the Natura 2000 database. The dataset NATURA2000SITES enclosed information on site code and type as well as area, dataset HABITATS included information, for example, on site code, HD Annex I habitat code and habitat cover, while BIOREGION enclosed information on site code, and biogeographic region where the site is located (for detailed information see Appendix S1: Table S2). “Site type” depends on the directive that the site is designated under, for example, the Birds Directive (BD) (i.e., Special Protection Areas, SPAs), the HD (i.e., Sites of Community Importance, SCIs), and SACs (Appendix S1: Table S1). The NATURA2000SITES dataset consisted of 27,031 entries; we removed sites where the total area was missing or was <0.0001 ha, resulting in 26,925 sites. The HABITATS dataset had 152,750 entries; we discarded entries where data on habitat area were missing (e.g., all habitat entries from Natura 2000 sites in Croatia), or area was <0.0001 ha, resulting in 145,253 entries. After joining the two datasets based on the site code, we removed sites where the total area of habitats was >100.01% as well as sites designated only under the BD (i.e., SPAs whose boundaries do not overlap with SCIs/SACs) since HD Annex I protected habitats were not part of the designation. The resulting dataset included 22,073 sites and 132,646 habitat entries (Appendix S1: Table S2).

**FIGURE 1:**
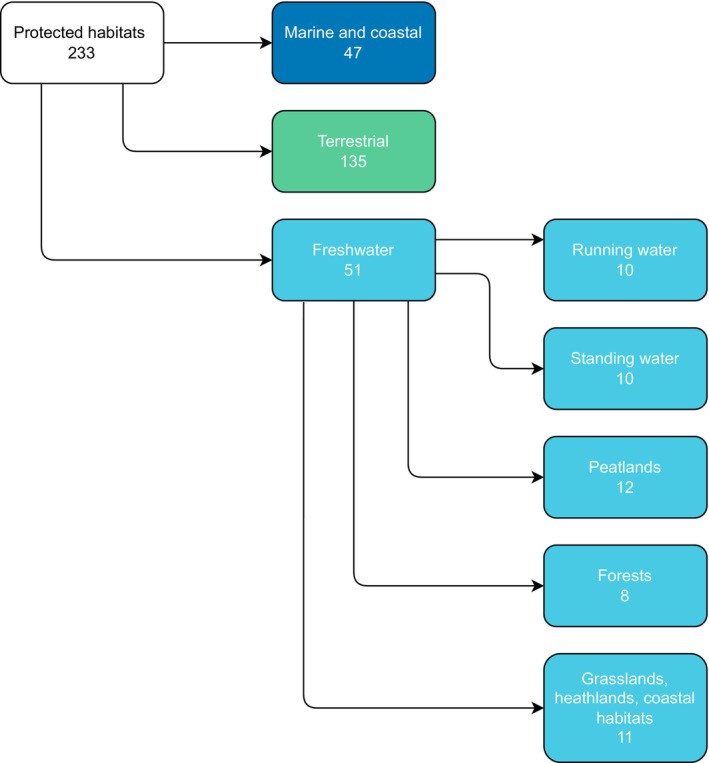
Habitats protected under the EU Habitats Directive Annex I, categorized into three habitat classes and further into one of the five freshwater habitat classes.

For the analyses, we created an additional dataset (Freshwater, marine and coastal, and terrestrial habitats; Appendix S1: Table S2). First we assigned a habitat class to each of the HD Annex 1 listed habitats in the HABITATS dataset: (1) freshwater, (2) marine and coastal, and (3) terrestrial) (233 in total; Appendix S1: Table S3; Figure 1). Freshwater habitat types were broadly defined and extended the definition of EEA (2020); we thus included not only freshwater habitats in the strict sense (codes 3xxx in the HD Annex I) but also GWDTE, whose water requirements are protected by the WFD Article 1 implying that bog, mire, fen, wet forest, certain types of scrub, natural, and semi‐natural grassland habitats were treated as freshwater habitats (Appendix S1: Table S3). Then, we created a dataset containing only freshwater habitats (Freshwater habitats; Appendix S1: Table S2), where each freshwater habitat type was assigned to one of the five groups: (1) running (lotic) or (2) standing (lentic) water habitats, (3) peatlands, (4) forests, or (5) grasslands, heathlands, and coastal habitats (Figure 1). Two habitat types, sub‐Mediterranean grasslands of the *Molinio‐Hordeion secalini* (Habitat code 6540) and Tufa cascades of karstic rivers of the Dinaric Alps (32A0*), were found only in Croatia and, due to missing data of this country, were not represented in the final dataset. All freshwater habitat types (55,525 entries in total) were further assigned to one of the following nutrient level classes: oligo‐, meso‐, or eutrophic, based on habitat descriptions available in the European Nature Information System (EUNIS) (EEA, 2024a). If these habitat descriptions did not enclose information on nutrient levels, we consulted the European RL of Habitats (Eionet, 2024) or vegetation descriptions (Chytrý et al., 2024).

Data on biogeographic regions for the Freshwater habitats were obtained from the dataset BIOREGION. The BIOREGION dataset included both terrestrial (i.e., Alpine, Atlantic, Black Sea, Boreal, Continental, Macaronesian, Mediterranean, Pannonian, Steppic) and marine biogeographic regions (Marine Atlantic, Marine Baltic, Marine Black Sea, Marine Macaronesian, and Marine Mediterranean). We confined the number of regions to the six main terrestrial biogeographic regions in continental Europe: Alpine, Atlantic, Boreal, Continental, Mediterranean, and Pannonian (Appendix S1: Table S4). The remaining regions were excluded because they either cover only a small area (e.g., the Macaronesian region covers only up to 0.3% of the EU area) or primarily represent marine habitats. The BIOREGION dataset was incomplete, however, with missing region assignment for some Natura 2000 sites, incorrect percentage values (e.g., 0% instead of 100% for sites fully within a region), or cases where sites spanned multiple regions. To ensure data reliability, we included only those sites where at least 70% of the area fell within a single region, as well as sites where the highest reported percentage was 100% (e.g., BE2200037, which belongs to the Atlantic biogeographic region, but was incorrectly recorded as 0%). Subsequently, we merged the filtered biogeographic region dataset with the “Freshwater habitat dataset,” based on the Natura 2000 site code. After visual inspection of sites and biogeographic regions, we corrected the 0% values to 100% for 373 sites, except for BE2200036, where we adjusted the value to 79% (i.e., 79% of the area is in the Atlantic biogeographic region and the remainder in the Continental region). Habitat entries without biogeographic regions or the percentage lower than 70% were labeled as “NA” (7239 entries).

Data on habitat distribution within the Natura 2000 network were then compared with the geographical coverage of the habitats across Europe according to the European RL of Habitats (Eionet, 2024), which uses the same terrestrial biogeographic regions as included in the BIOREGION dataset. The European RL covers natural and semi‐natural habitat types and provides an assessment of the risk of collapse of these habitat types based on a consistent set of criteria and categories as well as detailed data and expertise (Janssen et al., 2016). The RL habitats has been cross‐linked with Annex I habitats of the HD (Directive 92/43/EC) using five different classification categories (EEA, 2024b; Appendix S1: Table S4). The crosslinks were expert‐based interpretations established to identify ecological overlap in the various definitions used. To ensure full biogeographic coverage of the HD Annex I habitats, we applied an inclusive cross‐linking approach, which may broaden the inferred biogeographic distribution for some of the habitats. We included habitats where the HD Annex I and RL habitat definitions were identical (e.g., HD Annex I habitat *Natural dystrophic lakes* [*3160*] and ponds is equal to RL habitat *C1.4 Permanent dystrophic waterbody*), closely matched (i.e., more or less equal as in case of habitat *Southern riparian galleries and thickets* [*Nerio‐Tamaricetea and Securinegion tinctoriae*] and *F9.3 Mediterranean riparian scrub* [*92D0*]), or where either the RL habitat or the HD Annex I habitat was defined broader (e.g., RL habitat *C1.6a Temperate temporary waterbody is defined broader* than habitat *Lakes of gypsum karst* [*3190*]). If a HD Annex I habitat corresponded to multiple RL habitats, we incorporated data from all relevant biogeographic regions (i.e., habitat 3120 *Oligotrophic waters containing very few minerals generally on sandy soils of the West Mediterranean, with Isoetes spp* corresponds to two RL habitats: C1.1b, found in Atlantic and Boreal biogeographic regions, and C1.6B, found in Mediterranean biogeographic region; in this case, all three biogeographic regions were included). If the RL habitats were typical for one or more biogeographic regions, we used the indicated regions. If no specific region was indicated, we assumed that the RL habitat occurs in all biogeographic regions (Appendix S1: Table S4). Due to lack of data on the distribution on some RL habitats, we also considered low‐importance crosslinks (i.e., cases where the relationship was insignificant, i.e., low‐importance relationship, and affected only a small part of the particular EU HD I habitat). For coastal and halophytic habitats, that is, Estuaries (1130) and Boreal Baltic inlets (1650), which were linked to >20 RL habitats each, we aggregated biogeographic region data based on the relevant terrestrial regions mentioned above. Habitats found in the Baltic Sea region (e.g., Boreal Baltic inlets (1650)) were included in both the boreal and continental biogeographic regions (Appendix S1: Table S4).

Data analysis was done using R version 4.4.1 (R Core Team, 2024).

To make the data on protected freshwater habitats within the Natura 2000 network easily accessible for stakeholders, for example, the EU institutions, member states, and local managers, we developed the Merlin webtool (https://www.waterwebtools.com/merlin). The webtool provides a general overview of freshwater, terrestrial, and marine habitats in the Natura 2000 network with information on, for example, coverage of the different habitat types and their conservation status with a possibility to visit individual Natura 2000 sites to extract information on protected habitats and species designated within each site. Additionally, we have developed a search function that can be used within the tool that allows the user to easily get an overview of, for example, sites with a specific protected habitat and/or species that can facilitate the identification of underrepresented habitats in a specific region. Videos explaining how to use the tool are available on the MERLIN webtool landing page.

## RESULTS

### Natura 2000 network

**FIGURE 2:**
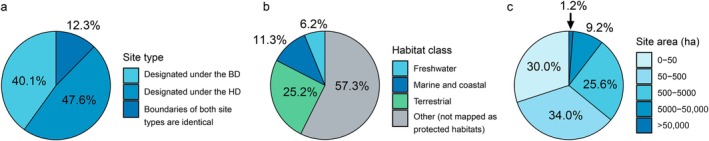
The coverage of Natura 2000 site types designated under the Habitats Directive, under both the Birds and Habitats Directives (i.e., where the boundaries of both site types are identical, and where these are not identical) (a). The coverage of protected freshwater, marine and coastal, and terrestrial habitats within the Natura 2000 network, and area not mapped as protected habitats (b). The size of Natura 2000 sites (in hectares) categorized into different size classes (c). BD, Birds Directive; HD, Habitats Directive.

Sites designated under the BD (SPAs) cover 40.1% of the total area of the Natura 2000 network, and sites designated under the HD (SCIs/SACs) cover 47.6% of the total area, while the borders of SPAs and SCIs/SACs overlap in 12.3% of the network (Figure 2a). Since protected habitats were not designated under the BD, we focused our analyses on sites designated under the HD (i.e., SCIs/SACs) and sites where boundaries of SPAs and SCIs/SACs overlap (in the following, these areas will be referred to as areas designated under the HD).

Overall, protected habitats cover 42.7% of the total area designated under the HD, while the remaining 57.3% were not mapped as protected habitats (Figure 2b). Of the total area of protected habitats, 25.2% are terrestrial, 11.3% marine and coastal, and 6.2% freshwater (Figure 2b).

**FIGURE 3:**
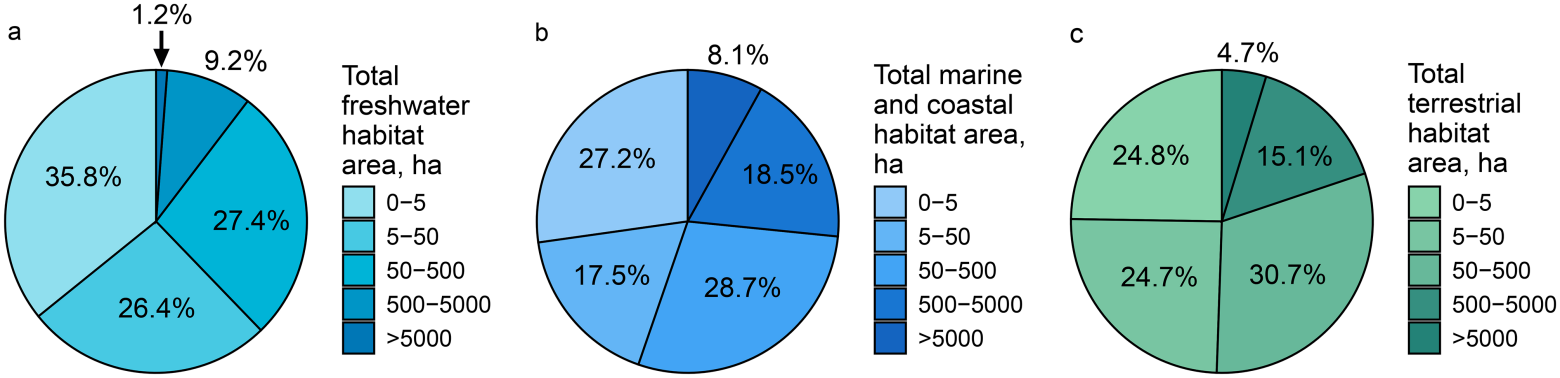
Size distribution of areas with freshwater (a) represented in 15,072 Natura 2000 sites with a total area 5,476,383 ha; marine/coastal (b) represented in 3302 sites, 10,049,546 ha; and terrestrial habitats (c) represented in 16,922 sites, 22,488,297 ha in Natura 2000 sites designated under the Habitats Directive and those designated under both the Birds and Habitats Directives (i.e., where the boundaries of both site types are identical).

The size of Natura 2000 sites designated under the HD varies greatly, ranging from 0.01 to 5,899,544.0 ha. However, the majority of sites are small, with 30% being smaller than 50 ha and 34% being between 50 and 500 ha (Figure 2c). Marine and coastal habitats, as well as terrestrial habitats, tend to cover large areas, with approximately 25% of sites exceeding 500 ha (Figure 3b,c) compared to freshwater habitats with only 10.4% exceeding 500 ha (Figure 3a).

### Freshwater habitat types

**FIGURE 4:**
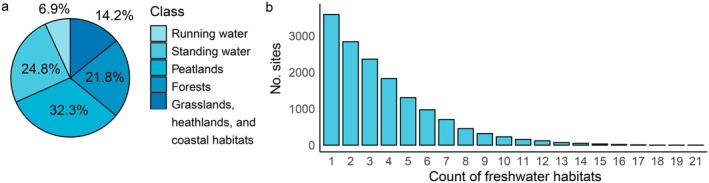
Distribution of freshwater habitat classes (a) and count of freshwater habitats (b) in Natura 2000 in sites designated under the Habitats Directive and those designated under both the Birds and Habitats Directives (i.e., where the boundaries of both site types are identical).

**FIGURE 5:**
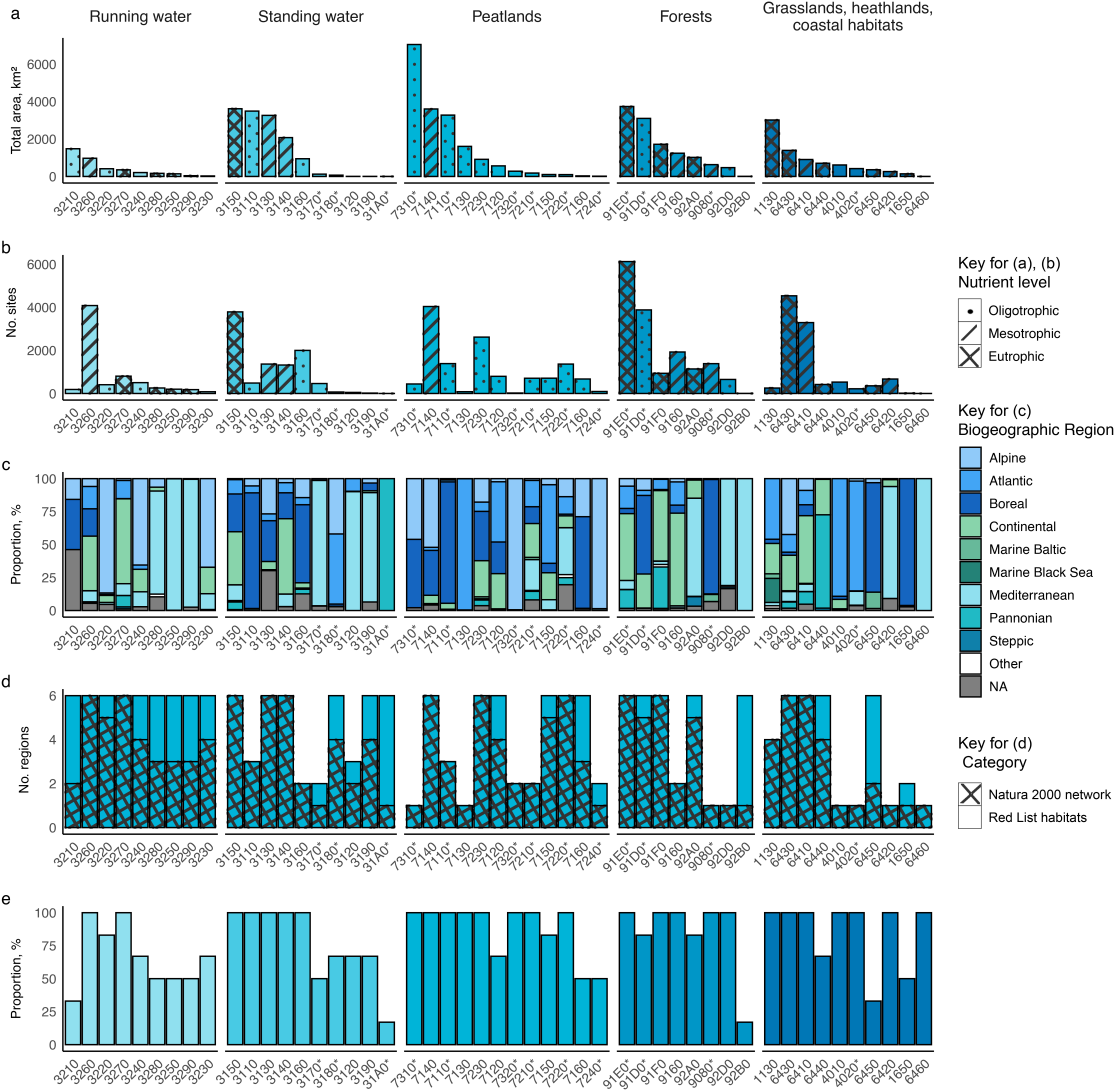
Overview of freshwater habitats. See Appendix S1: Section S1 for habitat codes for each habitat class. (a) Total area of different habitat types with indication of nutrient level. (b) Number of sites with different habitat types with indication of nutrient level. (c) Distribution of habitats in terrestrial and marine biogeographic regions, in category “Other” Black Sea, Macaronesian, Marine Atlantic, and Marine Mediterranean biogeographic regions are joined. (d) Number of terrestrial biogeographic regions covered by Natura 2000 network and European Red List habitats. (e) Proportion of terrestrial biogeographic regions represented in Natura 2000 network and overlapping with European Red List habitats in Natura 2000 sites designated under the Habitats Directive and those designated under both the Birds and Habitats Directives (i.e., where the boundaries of both site types are identical).

The coverage of different types of freshwater habitats within the Natura 2000 network varies (Figure 4): 32.3% are peatlands, 24.8% standing water habitats, 21.8% forests, and only 6.9% running water habitats (Figure 4a). Freshwater habitats with the largest coverage were Aapa mires (Habitat code 7310*) covering 704,600 ha corresponding to 12.9% of total area of all freshwater habitats, Alluvial forests with *Alnus glutinosa* and *Fraxinus excelsior* (91E0*) covering 373,450 ha corresponding to 6.8%, and Natural eutrophic lakes with *Magnopotamion‐* or *Hydrocharition*‐type vegetation (3150) covering 361,440 ha and corresponding to 6.6%. Habitats with the lowest coverage were Lakes of gypsum karst (3190; 240 ha; Figure 5a), Peat grasslands of Troodos (6460; 4 ha), and Transylvanian hot‐spring lotus beds (31A0*; 0.02 ha; Figure 5a).

In total, 15,072 Natura 2000 sites include at least one protected freshwater habitat type and more than two‐thirds (70.5%) include one to four habitat types (Figure 4b). Areas with the highest number of freshwater habitat types are situated in Latvia (LV0200100, Gaujas nacionālais parks with 21 habitat types and LV0200200, Ķemeru nacionālais parks with 19 habitat types) and France (FR2500081, Havre de Saint‐Germain‐sur‐Ay et Landes de Lessay with 18 habitat types).

The number of Natura 2000 sites where different habitat types occur varies considerably (Figure 5b). The most common freshwater habitats were Alluvial forests with *A. glutinosa* and *F. excelsior* (91E0*; 6124 Natura 2000 sites), Hydrophilous tall herb fringe communities of plains and montane to alpine levels (6430; 4535 sites), and water courses of plain to montane levels with the *Ranunculion fluitantis* and *Callitricho*‐*Batrachion* vegetation (3260; 4082 sites). In contrast, the forest habitat Riparian formations on intermittent Mediterranean water courses with *Rhododendron ponticum*, *Salix*, and others (92B0) is represented in five sites only, while Peat grasslands of Troodos (6460) and Transylvanian hot‐spring lotus beds (31A0*) are present in only one Natura 2000 site each.

Analyzing the nutrient status of the different habitat types categorized into oligotrophic habitats being characterized by low, mesotrophic by intermediate, and eutrophic by relatively high nutrient levels reveals that nutrient‐rich habitats are slightly better represented in the Natura 2000 network. Specifically, eutrophic habitat types are present in 19,003 sites, mesotrophic in 18,436 sites, and oligotrophic in 18,086 sites. Some of the most widely represented eutrophic habitat types are Alluvial forests with *A. glutinosa* and *F. excelsior* (91E0*), Hydrophilous tall herb fringe communities of plains and montane to alpine levels (6430), and Natural eutrophic lakes with *Magnopotamion‐* or *Hydrocharition*‐type vegetation (3150; Figure 5b). However, the total area of oligotrophic habitat types is higher (46.5%) than the total area of mesotrophic and eutrophic habitats (23.6% and 29.9%, respectively). Two out of eight habitat types with the highest total area are eutrophic habitats (Alluvial forests with *A. glutinosa* and *F. excelsior* [91E0*] and natural eutrophic lakes with *Magnopotamion‐* or *Hydrocharition*‐type vegetation [3150]; Figure 5a).

Many habitats are found in only a few Natura 2000 sites but cover a large proportion, while others are widely distributed but cover relatively smaller areas. This is evident when comparing the number of sites with the coverage of the specific habitat type (Figure 5a,b). For example, the running water habitat type water courses of plain to montane levels with the *R. fluitantis* and *Callitricho*‐*Batrachion* vegetation (3260) is distributed in 4082 different Natura 2000 sites but covers only 1.8% (97,106 ha) of the total freshwater habitat area, thus showing a limited spatial extent despite broad representation. In contrast, the total area of, for example, peatland habitat Aapa mires (7310*) covers 12.9%, but is restricted to just 441 sites.

### Freshwater habitat distribution across biogeographic regions

The spatial representation of freshwater habitat types in the Natura 2000 network varies significantly across biogeographic regions. Some habitats are confined to a single region, for example, Transylvanian hot‐spring lotus beds (31A0*; Pannonian biogeographic region), Peat grasslands of Troodos (6460; Mediterranean region), and Riparian formations on intermittent Mediterranean water courses with *R. ponticum*, *Salix*, and others (92B0; Mediterranean region). In contrast, other habitats are more widely distributed in the network. For example, Alpine rivers and the herbaceous vegetation along their banks (3220) occur in five biogeographic regions, Natural dystrophic lakes and ponds (3160) in eight, and Riparian mixed forests of *Quercus robur*, *Ulmus laevis*, and *Ulmus minor*, *F. excelsior*, or *Fraxinus angustifolia* along the great rivers (*Ulmenion minoris*) (91F0) in nine (Figure 5c).

Some habitats are well represented across their natural geographical range within the Natura 2000 network, whereas others are less well represented (Figure 5d). Notably, 2 out of 9 running water habitats, 5 out of 10 standing water habitats, 8 out of 12 peatland habitats, and 5 out of 8 forest habitats are represented in the Natura 2000 network across all biogeographic regions where they naturally occur (Figure 5d,e). Here it should be noted, however, that for some sites and consequently also habitats within the sites, information on biogeographic region was not available, and additionally, that we applied an inclusive cross‐linking approach, which may broaden the inferred biogeographic distribution for some of the habitats (Figure 5c).

### Natura 2000 webtool

The Merlin webtool has been developed as part of this study to display the data underlying the analyses. The webtool allows the user to explore, select, and extract data for further analyses on freshwater habitats and species within the EU. The webtool can be accessed at https://www.waterwebtools.com/merlin.

The webtool provides different options to look closer into the Natura 2000 network. The different options can be selected by ticking various layers on and off as detailed out in S4. The layers include, for instance, an overview at the EU level showing the fraction of freshwater‐related habitats in the Natura 2000 network scaled in percentages of the total area for each of the individual sites, and the fraction of freshwater habitats in excellent conservation status again at the EU level and scaled in percentages of the total area for each of the individual sites. These options are explained more in depth in Appendix S1: Section S2. Additionally, videos explaining how to use the tool are available from the webtool landing page.

Additionally, the webtool allows the user to click on a specific Natura 2000 site on the map and go to the Report View to get an overview of HD Annex I listed habitats, categorized into freshwater‐related, terrestrial, marine, and coastal habitats, to explore the coverage of specific habitats and to identify species designated for the site (HD Annexes II, IV listed species and BD Annex I listed species). Again this option is explained in more detail in Appendix S1: Section S2 and in the videos provided on the MERLIN webtool landing page.

Equally important, the webtool also allows the user to select and extract data from the webtool using the Search Function. If, for example, a user has a specific interest in identifying Natura 2000 sites with a specific habitat type, for instance “Water courses of plain to montane levels with the *R. fluitantis* and *Callitricho‐Batrachion* vegetation” within the whole network or within a certain region, it is possible to extract these data and to export them in different formats. This option is also explained in more detail in Appendix S1: Section S2 and in videos hosted in the MERLIN webtool.

## DISCUSSION

Natura 2000 is the largest coordinated network of nature conservation areas globally (EEA, 2020), but our findings display that freshwater habitats are represented with a low coverage within the network. Although we found that Annex I listed freshwater habitats cover 6.2% of the total area of habitats designated under the HD within the Natura 2000 network when including GWDTE, protected by the WFD's Article 1, this corresponds to only 2.7% of the total area of the Natura 2000 network. In comparison, a rough estimate from Corine Land Cover (CLC) reveals that approximately 6.3% of European land area is covered by running and standing waters (including CLC classes 511, 512, 411, 412; EEA, 2020), a number, however, that underestimates the total area as, for example, only running waters with a width of at least 100 m are included in CLC 511 due to the spatial resolution of CLC (European Environment Agency, 2018). Furthermore, wet forests, which comprise approximately 22% of the total freshwater habitat area within the Natura 2000 network, and grasslands, heathlands, and coastal habitats, which comprise approximately 14.2% of the total freshwater habitat area within the network are not included in the total percentage of 6.3%. Thereby, the coverage of freshwater habitats is at a minimum more than twice as high outside the Natura 2000 network compared to inside the network, lending support to the first hypothesis that freshwater habitats are not well represented in the Natura 2000 network.

Additionally, the Natura 2000 network does not provide a balanced representation of the various freshwater habitat types. When considering only standing and running waters, these habitat types cover 0.02 million km^2^ within the Natura 2000 network, whereas their total coverage across the EU is 0.13 million km^2^ (EEA, 2020). Running water habitats in particular are underrepresented, accounting for only 0.4% of the overall Natura 2000 network area, compared to lakes and peatlands accounting for 1.4% and 1.9% of the total network area, respectively. While the inherently linear nature of running water systems may partly explain their limited spatial extent within the network, this characteristic alone does not sufficiently account for their disproportionately low representation. For example, Denmark's stream network spans approximately 30,000 km, yet only 86 km—equivalent to 9 km^2^—have been designated as running water habitats within the Natura 2000 network, amounting to just 0.3% of the national network (Fredshavn et al., 2019; Levin, 2019).

The low coverage of running water habitats within the Natura 2000 network is concerning. These habitats support a very high number of species including approximately 71% of Europe's threatened freshwater species (Sayer et al., 2025), which is largely driven by a pronounced physical variability associated with spatial and temporal fluctuations in temperature, discharge, connectivity, and habitat (Bunn & Arthington, 2002). The limited extent of designated running water habitats within the network can be critical for supporting species dispersal, recolonization, and long‐term persistence, in particular because running waters exhibit high levels of local endemism and species turnover (β‐diversity) among catchments, meaning that species loss from a single river system may lead to global extinction, particularly in biodiversity hotspots such as the Mediterranean (Dudgeon, 2019; Fox & Magoulick, 2024). Low spatial coverage of running water habitats within individual Natura 2000 sites therefore exacerbates extinction risk, as it undermines the ecological resilience typically provided by a dense and structurally complex river network with multiple confluences and branching patterns (Grantham et al., 2019; van Looy et al., 2019).

In addition to a low spatial coverage of freshwater habitats in the Natura 2000 network, the analyses also indicated that many freshwater habitats listed in Annex I are not represented across their full natural geographical range size thereby supporting the second hypothesis. For example, Alpine rivers and their ligneous vegetation with *Myricaria germanica* (3230) are represented in four out of six regions; Mediterranean temporary ponds (3170), being a priority habitat in Annex I, are represented in one of the two biogeographic regions that represent the natural range of this habitat type; and Fennoscanican natural rivers (3210) are represented in two out of the six biogeographic regions. In contrast, some eutrophic habitats, for example, Alluvial forests and Natural eutrophic lakes, are well represented both concerning total protected area, number of sites, and their distribution across all relevant biogeographic regions. Other habitat types, for example, water courses of plain to montane levels with the *R. fluitantis* and *Callitricho‐Batrachion* vegetation (3260) are also well represented in the network, but with minimal coverage as mentioned above. It should be noted that, due to the methodology used, particularly the inclusion of low‐importance crosslinks and assumptions about unspecified regional occurrence, the regional coverage may be somewhat overestimated. This conservative approach was adopted to ensure that all potential natural regions are considered, but results should as a consequence be interpreted as indicative rather than definitive for exact biogeographical representation.

One argument for the limited representation of freshwater habitats in Natura 2000 could be that rivers, lakes, and groundwater‐dependent vegetation are also protected under the WFD and therefore with less need to be covered by the Natura 2000 network. While the WFD and the HD overlap in the ecosystems they cover, achieving or maintaining good ecological status under the WFD is not the same as achieving or maintaining favorable conservation status under the HD. Favorable conservation status requires that the natural range and area that a habitat occupies is stable or increasing, that the structure and functions of the habitat are maintained, and that the conservation status of its typical species is favorable. On the other hand, the primary goal of the WFD is to ensure the protection and sustainable management of water resources across Europe, with the overarching goal of achieving good ecological and chemical status in surface water bodies and good quantitative and qualitative status of groundwater bodies. According to WFD Annex V, the good quantitative status of a groundwater body requires that groundwater levels remain unaffected by anthropogenic alterations that could damage GWDTE. Thereby the WFD does not comprehensively address all aspects of biodiversity nor is its monitoring network designed to assess changes in biodiversity (Argillier & Lepage, 2011). As an example, a recent study revealed that ecological status is not a reliable indicator of freshwater biodiversity in Danish rivers (Baattrup‐Pedersen et al., 2023; Henriksen et al., unpublished manuscript). Thus, sites with high ecological status often lack declining and rare species, and disappearance of such species did not necessarily lead to a downgrading of ecological status. Consequently, it is possible to maintain a high ecological status at a river site even if declining and rare species disappear.

Based on the findings presented here, we question whether the EU NRR can substantially contribute to halting freshwater biodiversity loss with a prioritization of restoration efforts within the existing boundaries of the Natura 2000 network. The current spatial coverage of freshwater habitats in the network is limited and, additionally, our results indicate that it does not adequately encompass the natural range of several habitat types, which may constrain their capacity to meet key spatial prerequisites associated with the long‐term persistence of their typical species. We therefore argue that a systematic strengthening of the Natura 2000 network is required, with particular emphasis on: (1) expanding the extent of designated freshwater habitat types both in‐ and outside the existing network if necessary to adequately cover freshwater habitats, and (2) identifying and addressing representation gaps across all relevant biogeographic regions.

To support this process at the member state level, we have developed the Merlin webtool, which enables relevant stakeholders including EU institutions, national agencies, and local managers to examine the distribution of freshwater habitats and species listed under Annexes I, II, and IV of the HD and Annex I of the BD within Natura 2000 sites. The tool provides access to detailed information on habitat types, their coverage, and conservation status at multiple spatial scales (e.g., site and national levels). This functionality facilitates assessments of whether improved representation of freshwater biodiversity can be achieved by designating additional habitat types within existing Natura 2000 sites at the member state level (57.3% of the area designated under the HD is not currently mapped as protected habitats) or whether the identification and inclusion of new sites is necessary.

Additionally, to effectively address the decline of freshwater biodiversity, a closer coordination between monitoring within the framework of the WFD and HD is essential. These two frameworks currently operate with differing methodologies, targets, and governance mechanisms, but integration of data on the biological quality elements (sensu WFD) and protected habitats and species (sensu HD) should serve as a basis for management decisions. For example, re‐establishing natural flow regimes, reconnecting floodplains, and removing migration barriers could benefit both biological quality elements and the conservation status of freshwater habitats protected under the HD (Arthington et al., 2024; Haase et al., 2025; Thieme et al., 2023). At the same time, monitoring should also be in place to assess the effectiveness for both directives, including monitoring that addresses multiple stressors and interactions within freshwater ecosystems (Reid et al., 2019; Tickner et al., 2020) and multiple dimensions of biodiversity (species richness, functional diversity, rarity). A fuller exploration of how closer coordination between WFD and HD monitoring could be operationalized, including harmonization of indicators, spatial scales, and assessment cycles, is clearly warranted, but lies beyond the scope of the present study. Nevertheless, highlighting this need underscores the importance of aligning policy frameworks to support more effective freshwater restoration and conservation. Furthermore, if the Natura 2000 network is to be effectively expanded to include and support running water habitats, a catchment‐based approach in selecting additional sites is essential. River systems function as interconnected networks, where upstream activities directly influence downstream habitat quality and ecological integrity. Protecting or designating isolated river sections without addressing broader catchment‐level pressures can undermine restoration outcomes. Thus, a catchment‐based perspective is necessary to preserve the ecological processes and connectivity essential for the survival of both typical and rare freshwater species, thereby aligning spatial planning with the natural functioning of freshwater ecosystems (Johnson et al., 2007; Schürings et al., 2024). Furthermore, this approach is likely to be particularly effective if including unmodified and free‐flowing rivers and their catchments, which can serve as refuges and source populations for colonization (van Rees et al., 2025).

### Conclusions

This study showed that freshwater ecosystems are not adequately represented within the Natura 2000 network. We found that freshwater habitats listed in the HD's Annex I cover 6.2% of the total area of Natura 2000 sites, even when including GWDTE protected by the WFD's Article 1. The calculated coverage thereby encompasses wetlands, wet forests, wet grasslands, heathlands, and coastal habitats in addition to running and standing waters. Additionally, the Natura 2000 network does not provide a balanced representation of the various freshwater habitat types either, with running water habitats being particularly underrepresented, accounting for only 0.4% of the overall Natura 2000 network area. In addition to low spatial coverage, the analyses indicated that several freshwater Annex I habitats are not represented across their full natural geographical range, pointing to limitations in a key spatial component of the favorable reference area concept. Based on the obtained results, we therefore argue that a systematic strengthening of the Natura 2000 network is required, with particular emphasis on: (1) expanding the extent of designated freshwater habitat types in‐ and outside the existing network if necessary to adequately cover freshwater habitats, and (2) identifying and addressing representation gaps across all relevant biogeographic regions. To support this process, we have developed a webtool based on the datasets underlying the analyses that enables relevant stakeholders—including EU institutions, national agencies, and local managers—to examine the distribution of freshwater habitats and species protected within Natura 2000 sites. The webtool can be accessed at https://www.waterwebtools.com/merlin.

## AUTHOR CONTRIBUTIONS

**Annette Baattrup‐Pedersen:** Writing—original draft; writing—review and editing; supervision; project administration; methodology; investigation; funding acquisition; formal analysis; conceptualization. **Marta Baumane:** Writing—original draft; writing—review and editing; visualization; validation; methodology; investigation; formal analysis; data curation. **Anders Nielsen:** Software; visualization; writing—review and editing. **Dennis Trolle:** Software; visualization; writing—review and editing. **Paulo Branco:** Writing—review and editing. **Florian Borgwardt:** Writing—review and editing. **Daniel Hering:** Writing—review and editing; funding acquisition. **Sebastian Birk:** Writing—review and editing; funding acquisition.

## CONFLICT OF INTEREST STATEMENT

The authors declare no conflicts of interest.

## Supporting information


Appendix S1.

## Data Availability

Data (Baattrup‐Pedersen et al., 2025) are available in Zenodo at https://doi.org/10.5281/zenodo.17601833.
