# Supplementary material for: Freshwater habitats within the Natura 2000 network
Source: Ecol Appl. 2026 Apr 22;36:e70241. doi: 10.1002/eap.70241 (PMC13101043; doi:10.1002/eap.70241)
Supplement: Supplementary file 1 — Appendix S1. [file EAP-36-e70241-s001.pdf]

Appendix S1

Freshwater abitats within the Natura 2000 network

Annette Baattrup-Pedersen, Marta Baumane, Anders Nielsen, Dennis Trolle, Paulo Branco, Florian Borgwardt, Daniel Hering, Sebastian Birk

Ecological Applications

Table S1

Overview of the legislation at the EU-level concerning freshwater habitats conservation and protection covered in the article.

Legislation	Description	Reference
Habitats Directive (Council Directive 92/43/EEC of 21 May 1992 on the conservation of natural habitats and of wild fauna and flora)	Annex I lists Natural habitat types of community interest whose conservation requires the designation of special areas of conservation (SACs)<br><br>Article 17 appoints EU Member States every six years to draw up a report on the implementation of the measures taken under the Habitats Directive	EC (1992)
Birds Directive (Directive 2009/147/EC of the European Parliament and of the Council of 30 November 2009 on the conservation of wild birds)	Article 12 appoints Member States every three years to report on the implementation of national provisions taken under this Directive<br><br>SPAs - special protection areas designated under the Birds Directive	EC (2009)
Water Framework Directive (Directive 2000/60/EC of the European Parliament and of the Council of 23 October 2000 establishing a framework for Community action in the field of water policy)	Article 1 states that the purpose of this Directive is to establish a framework for the protection of inland surface waters, transitional waters, coastal waters and groundwater	EC (2000)
Nature Restoration Regulation (Regulation (EU) 2024/1991 of the European Parliament and of the Council of 24 June 2024 on nature restoration and amending Regulation (EU) 2022/869)	Article 3 covers definitions used within the directive<br><br>Article 4 covers restoration of terrestrial, coastal and freshwater ecosystems	EC (2024)
European Red List of Habitats	The European Red List of Habitats provides an assessment of the risk of collapse of marine, terrestrial and freshwater natural and semi-natural habitats based on a consistent set of criteria and categories and detailed data and expertise	EEA (2024)

Table S2

Overview of datasets used in the data analysis.

Title	Description	Number of entries post data cleaning
Sites (EC, 2022; dataset “NATURA2000SITES”)	Site code (e.g., AT1101112)<br>Site type (A: Special Protection Areas (SPAs) designated under the Birds Directive, B: Sites of Community Importance (SCIs) or Special Areas of Conservation (SACs) designated under the Habitats Directive, C: boundaries of SPAs and SCIs/SACs are identical)<br>Area, ha (range 0.01, 7186094.0)	26925 sites
Freshwater, marine and coastal, and terrestrial habitats (EC, 2022, datasets “NATURA2000SITES” and “HABITATS”)	Site code (e.g., AT1101112)<br>Site type (B, C)<br>Area, ha (range 0.01, 5899544.0)<br>Habitat code (e.g., 3160)<br>Habitat class (Freshwater, marine and coastal, terrestrial)<br>Habitat cover, ha (range 0.01, 2267324.0)<br>Conservation (A: excellent; B: good; C: average or reduced, NA)	20073 sites, 132646 habitats
Freshwater habitats (EC, 2022, datasets “NATURA2000SITES”, “HABITATS”, and “BIOREGION”)	Site code (e.g., AT1101112)<br>Area, ha (range 0.1, 554732.0)<br>Habitat code (e.g., 3160)<br>Freshwater habitat class (Running water, standing water, peatlands, forests, other)<br>Habitat cover, ha (range <0.01, 64000.0)<br>Conservation (A: excellent; B: good; C: average or reduced, NA)<br>Nutrient status (oligo-, meso-, eutrophic, see Methods for detailed information)<br>Biogeographic region (e.g., Atlantic, Boreal)<br>Percentage of biogeographic region (range 70, 100; NA for 1936 site entries)	15072 sites, 55525 habitats

Table S3

Overview of analyzed freshwater habitats protected under the Habitats Directive Annex I (EC, 1992). * - priority habitats.

Code	Habitat
	**Coastal and halophytic habitats**
1130	Estuaries
1650	Boreal Baltic narrow inlets
	**Freshwater habitats**
3110	Oligotrophic waters containing very few minerals of sandy plains (*Littorelletalia uniflorae*)
3120	Oligotrophic waters containing very few minerals generally on sandy soils of the West Mediterranean, with *Isoetes* spp.
3130	Oligotrophic to mesotrophic standing waters with vegetation of the *Littorelletea uniflorae* and/or of the *Isoëto-Nanojuncetea*
3140	Hard oligo-mesotrophic waters with benthic vegetation of *Chara* spp.
3150	Natural eutrophic lakes with *Magnopotamion* or *Hydrocharition* — type vegetation
3160	Natural dystrophic lakes and ponds
3170*	Mediterranean temporary ponds
3180*	Turloughs
3190	Lakes of gypsum karst
31A0*	Transylvanian hot-spring lotus beds
3210	Fennoscandian natural rivers
3220	Alpine rivers and the herbaceous vegetation along their banks
3230	Alpine rivers and their ligneous vegetation with *Myricaria germanica*
3240	Alpine rivers and their ligneous vegetation with *Salix elaeagnos*
3250	Constantly flowing Mediterranean rivers with *Glaucium flavum*
3260	Water courses of plain to montane levels with the *Ranunculion fluitantis* and *Callitricho-Batrachion* vegetation
3270	Rivers with muddy banks with *Chenopodion rubri* p.p. and *Bidention* p.p. vegetation
3280	Constantly flowing Mediterranean rivers with *Paspalo-Agrostidion* species and hanging curtains of *Salix* and *Populus alba*
3290	Intermittently flowing Mediterranean rivers of the *Paspalo-Agrostidion*
32A0[1]	Tufa cascades of karstic rivers in the Dinaric Alps
	**Temperate heath and scrub**
4010	Northern Atlantic wet heaths with *Erica tetralix*
4020*	Temperate Atlantic wet heaths with *Erica ciliaris* and *Erica tetralix*
**NATURAL AND SEMI-NATURAL GRASSLAND FORMATIONS**	
6410	*Molinia* meadows on calcareous, peaty or clayey-silt-laden soils (*Molinion caeruleae*)
6420	Mediterranean tall humid grasslands of the *Molinio-Holoschoenion*
6430	Hydrophilous tall herb fringe communities of plains and of the montane to alpine levels
6440	Alluvial meadows of river valleys of the *Cnidion dubii*
6450	Northern boreal alluvial meadows
6460	Peat grasslands of Troodos
6540[1]	Sub-Mediterranean grasslands of the *Molinio-Hordeion secalini*

Code	Habitat
	**Raised bogs, mires and fens**
7110*	Active raised bogs
7120	Degraded raised bogs still capable of natural regeneration
7130	Blanket bogs (* if active bog)
7140	Transition mires and quaking bogs
7150	Depressions on peat substrates of the *Rhynchosporion*
7160	Fennoscandian mineral-rich springs and springfens
7210*	Calcareous fens with *Cladium mariscus* and species of the *Caricion davallianae*

7220*	Petrifying springs with tufa formation (*Cratoneurion*)
7230	Alkaline fens
7240*	Alpine pioneer formations of *Caricion bicoloris-atrofuscae*
7310*	Aapa mires
7320*	Palsa mires
	**Forests**
9080*	Fennoscandian deciduous swamp woods
9160	Sub-Atlantic and medio-European oak or oak-hornbeam forests of the *Carpinion betuli*
91D0*	Bog woodland
91E0*	Alluvial forests with *Alnus glutinosa* and *Fraxinus excelsior* (*Alno-Padion*, *Alnion incanae*, *Salicion albae*)
91F0	Riparian mixed forests of *Quercus robur*, *Ulmus laevis* and *Ulmus minor*, *Fraxinus excelsior* or *Fraxinus angustifolia*, along the great rivers (*Ulmenion minoris*)
92A0	*Salix alba* and *Populus alba* galleries
92B0	Riparian formations on intermittent Mediterranean water courses with *Rhododendron ponticum*, *Salix* and others
92D0	Southern riparian galleries and thickets (*Nerio-Tamaricetea* and *Securinegion tinctoriae*)

[1] – Not included in the final dataset due to missing habitat cover data from Croatia.

Table S4

Distribution of protected freshwater habitats in terrestrial biogeographic regions in Europe and comparison between natural distribution, based on European Red List of Habitats (RL; EEA, 2024), and distribution in Natura 2000 sites designated under the Habitats Directive (HD) and those designated under both the Birds Directive and the HD (i.e., where the boundaries of both site types are identical; EC, 2022). The relationship between the HD Annex I (EC, 1992) and RL habitats was described based on crosslinks (HD Annex I qualifier; EEA, 2024): = the type definitions are equal, ≈ more or less equal, < the type to the left of the qualifier is defined as a part of the type to the right of the qualifier, > the type to the left of the qualifier is defined as broader than and completely including the type to the right of the qualifier, # both types have a large part that overlaps, but also parts that do not overlap, * insignificant relationship, ** >20 RL habitats correspond to HD Annex I habitat. Abbreviations: AL – Alpine, AT – Atlantic, B – Boreal, C – Continental, M – Mediterranean, P – Pannonian biogeographic region. Biogeographic regions where the natural distribution of the habitat is not covered by the Natura 2000 network are highlighted in bold and gray.

			Biogeographic region							
**RL habitat code and name**	**HD Annex I qualifier**	**HD Annex I habitat code**	**ALP**	**ATL**	**BOR**	**CON**	**MED**	**PAN**	**Natural distribution (based on RL)**	**Distribution in Natura 2000 network**
**Running water habitats**										
C2.3 Permanent non-tidal, smooth-flowing watercourse	#*	3210	1	**1**	1	**1**	**1**	**1**	6	2
C2.2a Permanent non-tidal, fast, turbulent watercourse of montane to alpine regions with mosses	#	3220	1	1	1	1	1	**1**		
C3.5d Unvegetated or sparsely vegetated shore with mobile sediments in montane and alpine regions	#	3220	1	1	1	1	1		6	5
C2.2a Permanent non-tidal, fast, turbulent watercourse of montane to alpine regions with mosses	#*	3230	1	1	**1**	1	1	**1**		
C3.5d Unvegetated or sparsely vegetated shore with mobile sediments in montane and alpine regions	#*	3230	1	1	**1**	1	1			
F9.1 Temperate and boreal riparian scrub	>	3230	1	1	**1**	1	1	**1**	6	4
C2.2a Permanent non-tidal, fast, turbulent watercourse of montane to alpine regions with mosses	<*	3240	1	1	**1**	1	1	**1**		
C3.5d Unvegetated or sparsely vegetated shore with mobile sediments in montane and alpine regions	#*	3240	1	1	**1**	1	1		6	4

RL habitat code and name	HD Annex I qualifier	HD Annex I habitat code	Biogeographic region: ALP	ATL	BOR	CON	MED	PAN	Natural distribution (based on RL)	Distribution in Natura 2000 network
**Running water habitats**										
F9.1 Temperate and boreal riparian scrub	>*	3240	1	1	**1**	1	1	**1**		
C2.3 Permanent non-tidal, smooth-flowing watercourse	#	3250	1	**1**	**1**	1	1	**1**		
C3.5e Unvegetated or sparsely vegetated shore with mobile sediments in the Mediterranean region	#	3250					1		6	3
C2.2b Permanent non-tidal, fast, turbulent watercourse of plains and montane regions with Ranunculus spp.	#	3260	1	1	1	1	1	1		
C2.3 Permanent non-tidal, smooth-flowing watercourse	#	3260	1	1	1	1	1	1		
C2.4 Tidal river, upstream from the estuary	#*	3260		1						
C2.5a Temperate temporary running watercourse	#	3260			Unknown				6	6
C3.5a Periodically exposed shore with stable, eutrophic sediments with pioneer or ephemeral vegetation	#	3270	1	1	1	1	1	1	6	6
C3.5a Periodically exposed shore with stable, eutrophic sediments with pioneer or ephemeral vegetation	#	3280	1	**1**	**1**	1	1	**1**		
C3.5e Unvegetated or sparsely vegetated shore with mobile sediments in the Mediterranean region	#	3280					1		6	3
C3.5a Periodically exposed shore with stable, eutrophic sediments with pioneer or ephemeral vegetation	#	3290	**1**	**1**	**1**	1	1	**1**		
C3.5e Unvegetated or sparsely vegetated shore with mobile sediments in the Mediterranean region	#	3290					1		6	2

RL habitat code and name	HD Annex I qualifier	HD Annex I habitat code	Biogeographic region						Natural distribution (based on RL)	Distribution in Natura 2000 network
			ALP	ATL	BOR	CON	MED	PAN		
**Standing water habitats**										
C1.1a Permanent oligotrophic waterbody with very soft-water species	=	3110	1	1	1				3	3
C1.1b Permanent oligotrophic to mesotrophic waterbody with softwater species	>*	3120		1	**1**					
C1.6b Mediterranean temporary waterbody	>*	3120					1		3	2
C1.1b Permanent oligotrophic to mesotrophic waterbody with softwater species	#	3130		1	1					
C3.5b Periodically exposed shore with stable, mesotrophic sediments with pioneer or ephemeral vegetation	<	3130	1	1	1	1	1	1	6	6
C1.2a Oligotrophic to mesotrophic waterbody with Characeae	=	3140	1	1	1	1	1	1	6	6
C1.2b Mesotrophic to eutrophic waterbody with angiosperms	>	3150	1	1	1	1	1	1	6	6
C1.4 Permanent dystrophic waterbody	=	3160		1	1				2	2
C1.6b Mediterranean temporary waterbody	>	3170					1			
C3.5c Periodically exposed saline shore with pioneer or ephemeral vegetation	#	3170					1	**1**	2	1
C1.6a Temperate temporary waterbody	>	3180	1	1	1	1	**1**	**1**	6	4

RL habitat code and name	HD Annex I qualifier	HD Annex I habitat code	Biogeographic region ALP	ATL	BOR	CON	MED	PAN	Natural distribution (based on RL)	Distribution in Natura 2000 network
**Standing water habitats**										
C1.6a Temperate temporary waterbody	>	3190	1	**1**	1	1	1	**1**	6	4
C1.2b Mesotrophic to eutrophic waterbody with angiosperms	>*	31A0	**1**	**1**	**1**	**1**	**1**	1	6	1
**Peatland habitats**										
D1.1 Raised bog	#	7110		1	1					
D2.1 Oceanic valley bog	#*	7110		1						
D2.2b Relict mire of Mediterranean mountains	#*	7110					1		3	3
D1.1 Raised bog	#	7120		1	1					
G1.5 Broadleaved bog woodland on acid peat	#*	7120	1	1	1	1	**1**	**1**	6	4
D1.2 Blanket bog	=	7130		1					1	1
D2.1 Oceanic valley bog	#*	7140		1						
D2.2a Poor fen	#	7140	1	1	1	1	1	1		
D2.2b Relict mire of Mediterranean mountains	#*	7140					1			
D2.2c Intermediate fen and soft-water spring mire	#	7140	1	1	1	1	1	1		
D2.3a Non-calcareous quaking mire	<	7140	1	1	1	1	1	1		
D4.1c Calcareous quaking mire	#*	7140	1	1	1	1	1	1	6	6
D2.1 Oceanic valley bog	#	7150		1						
D.2.2a Poor fen	#	7150	1	1	1	1	1	**1**		
D2.2c Intermediate fen and soft-water spring mire	#	7150	1	1	1	1	1	**1**	6	5
C2.1a Base-poor spring and spring brook	>	7160	1	**1**	1	1	**1**	**1**		
D2.2c Intermediate fen and soft-water spring mire	#*	7160	1	**1**	1	1	**1**	**1**	6	3
D4.1b Tall-sedge base-rich fen	#	7210			1	1			2	2
C2.1b Calcareous spring and spring brook	>	7220	1	1	1	1	1	1	6	6
D4.1a Small-sedge base-rich fen and calcareous spring mire	<	7230	1		1	1				
D4.1c Calcareous quaking mire	#	7230	1	1	1	1	1	1	6	6
D4.2 Arctic-alpine rich fen	=	7240	1	**1**					2	1
D3.2 Aapa mire	=	7310			1				1	1
D3.1 Palsa mire	=	7320	1		1				2	2

RL habitat code and name	HD Annex I qualifier	HD Annex I habitat code	Biogeographic region						Natural distribution (based on RL)	Distribution in Natura 2000 network
			ALP	ATL	BOR	CON	MED	PAN		
**Forest habitats**										
G1.4 Broadleaved swamp woodland on non-acid peat	>	9080			1				1	1
G1.Aa Carpinus and Quercus mesic deciduous woodland	>	9160		1		1			2	2
G1.5 Broadleaved bog woodland on acid peat	<	91D0	1	1	1	1	**1**	1		
G3.Da Pinus mire woodland	<	91D0	1	1	1	1	**1**	1		
G3.Db Picea mire woodland	<	91D0	1	1	1	1	**1**	1	6	5
G1.1 Temperate and boreal softwood riparian woodland	#	91E0	1	1	1	1	1	1		
G1.2a Alnus woodland on riparian and upland soils	#	91E0	1	1	1	1	1	1	6	6
G1.2b Temperate and boreal hardwood riparian woodland	=	91F0	1	1	1	1	1	1	6	6
G1.1 Temperate and boreal softwood riparian woodland	#*	92A0	1	1	**1**	1	1	1		
G1.3 Mediterranean and Macaronesian riparian woodland	>	92A0	1	1	**1**	1	1	1	6	5
G1.3 Mediterranean and Macaronesian riparian woodland	>	92B0	**1**	**1**	**1**	**1**	1	**1**	6	1
F9.3 Mediterranean riparian scrub	≈	92D0					1		1	1
**Grassland, heathland, and coastal habitats**										
**	*	1130		1	1	1	1		4	4
**	*	1650			1	**1**			2	1
F4.1 Wet heath	>	4010		1					1	1
F4.1 Wet heath	>	4020		1					1	1
E3.5 Temperate and boreal moist or wet oligotrophic grassland	=	6410	1	1	1	1	1	1	6	6
B1.8b Mediterranean and Black Sea moist and wet dune slack	#*	6420					1			
E3.1a Mediterranean tall humid inland grassland	>	6420					1		1	1
E5.4 Moist or wet tall-herb and fern fringe of the lowlands	<	6430	1	1	1	1	1	1		
E5.5 Subalpine moist or wet tall-herb and fern fringe	<	6430	1						6	6

RL habitat code and name	HD Annex I qualifier	HD Annex I habitat code	Biogeographic region: ALP	ATL	BOR	CON	MED	PAN	Natural distribution (based on RL)	Distribution in Natura 2000 network
**Grassland, heathland, and coastal habitats**										
E3.4a Moist or wet mesotrophic to eutrophic hay meadow	>	6440	1	1	**1**	1	**1**	1	6	4
C5.2 Tall-sedge bed	#*	6450	1	**1**	1	**1**	**1**	**1**		
E3.4a Moist or wet mesotrophic to eutrophic hay meadow	#	6450	1	**1**	1	**1**	**1**	**1**	6	2
E3.1a Mediterranean tall humid inland grassland	>	6460					1		1	1

Section S1

Habitat codes for each habitat class. An asterisk denotes priority habitats.

Running water: 3210 Fennoscandian natural rivers, 3260 Water courses of plain to montane levels with the Ranunculion fluitantis and Callitricho-Batrachion vegetation, 3220 Alpine rivers and the herbaceous vegetation along their banks, 3270 Rivers with muddy banks with Chenopodion rubri p.p. and Bidention p.p. vegetation;

Standing water: 3150 Natural eutrophic lakes with Magnopotamion or Hydrocharition -type vegetation, 3110 Oligotrophic waters containing very few minerals of sandy plains (Littorelletalia uniflorae), 3130 Oligotrophic to mesotrophic standing waters with vegetation of the Littorelletea uniflorae and/or of the Isoeto-Nanojuncetea, 3140 Hard oligo-mesotrophic waters with benthic vegetation of Chara spp., 3160 Natural dystrophic lakes and ponds;

Peatlands: 7310 Aapa mires, 7140 Transition mires and quaking bogs, 7110* Active raised bogs, 7130 Blanket bogs, 7230 Alkaline fens, 7120 Degraded raised bogs still capable of natural regeneration, 7320 Palsa mires;

Forests: 91E0* Alluvial forests with Alnus glutinosa and Fraxinus excelsior (Alno-Padion, Alnion incanae, Salicion albae), 91D0* Bog woodland, 91F0 Riparian mixed forests of Quercus robur, Ulmus laevis and Ulmus minor, Fraxinus excelsior or Fraxinus angustifolia, along the great rivers (Ulmenion minoris), 9160 Sub-Atlantic and medio-European oak or oak-hornbeam forests of the Carpinion betuli, 92A0 Salix alba and Populus alba galleries, 9080* Fennoscandian deciduous swamp woods, 92D0 Southern riparian galleries and thickets (Nerio-Tamaricetea and Securinegion tinctoriae);

Grasslands, heathlands, and coastal habitats: 1130 Estuaries, 6430 Hydrophilous tall herb fringe communities of plains and of the montane to alpine levels, 6410 Molinia meadows on calcareous, peaty or clayey-silt-laden soils (Molinion caeruleae), 4010 Northern Atlantic wet heaths with Erica tetralix, 4020* Temperate Atlantic wet heaths with Erica ciliaris and Erica tetralix, 6440 Alluvial meadows of river valleys of the Cnidion dubii, 6450 Northern boreal alluvial meadows.

Section S2

Quick guide

Step by step manual displaying the various functions in the Merlin Webtool (2025).

1. The webtool can be accessed via this link: https://www.waterwebtools.com/merlin by clicking on "Open MERLIN app" or by scanning the QR code.

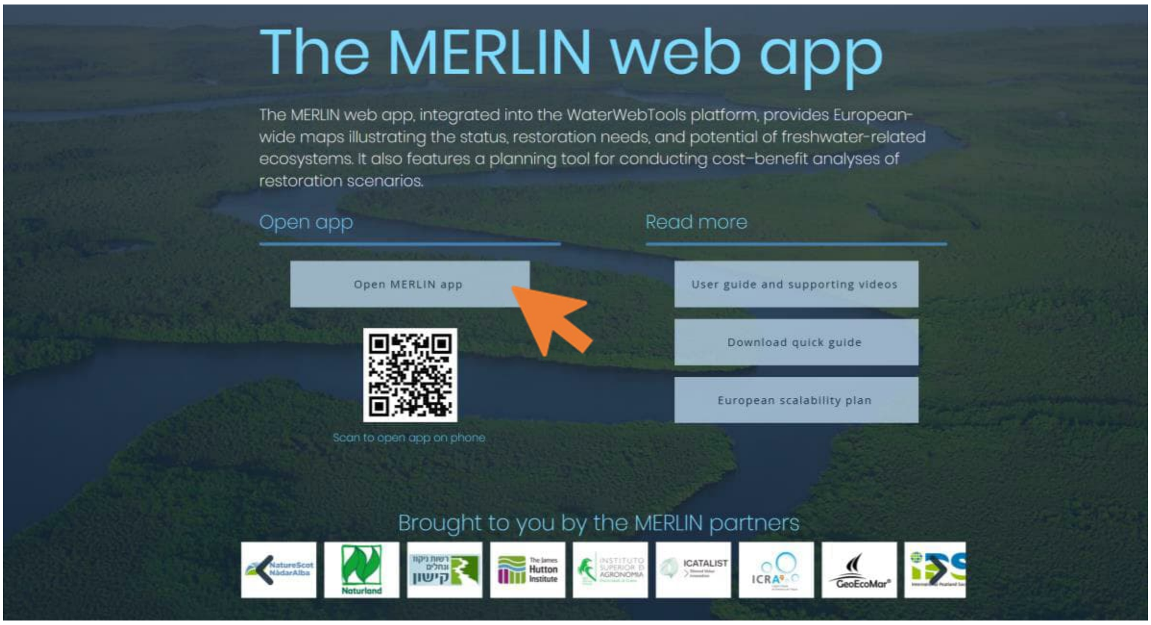

2. The user then has several options: changing the background map (1); choosing available layers in the Layers View (2); viewing legends in the Legend View (3); using the Map Search (4) to look for specific protected habitats, species, or Natura 2000 area; and downloading selected data or viewing a general overview of freshwater, terrestrial, marine and coastal habitats, and protected species in a specific Natura 2000 site in Report View (5).

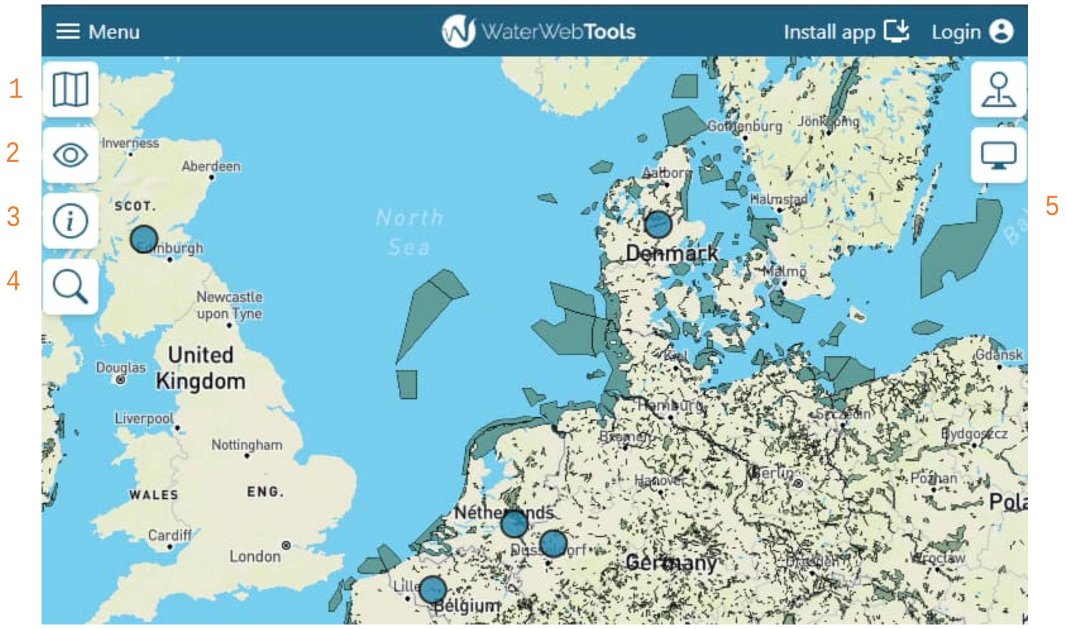

3. The webtool provides a comprehensive overview of freshwater habitat distribution and coverage within the Natura 2000 network. To access the maps, click on Layers View, then (1) Protected areas and EU legislation, then Habitats and species in Natura 2000 (Habitats Directive), and choose one of the available layers, e.g., the fraction of **freshwater-related habitats**. Other layers available for each Natura 2000 site include: **total area under protection**, **number of freshwater habitats**, **fraction of freshwater habitats in excellent conservation status**, and **data quality**.

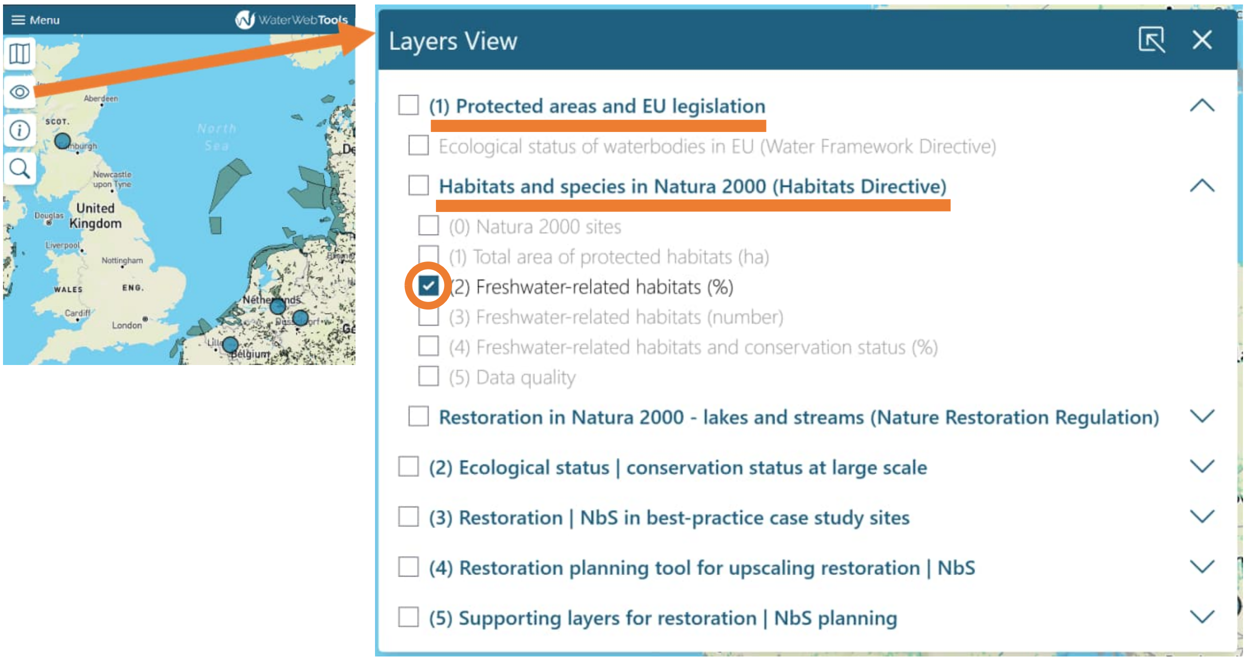

4. This is an example of a map showing the **fraction of freshwater-related habitats** in the Northern Zealand in Denmark. The legend is available under Legend View.

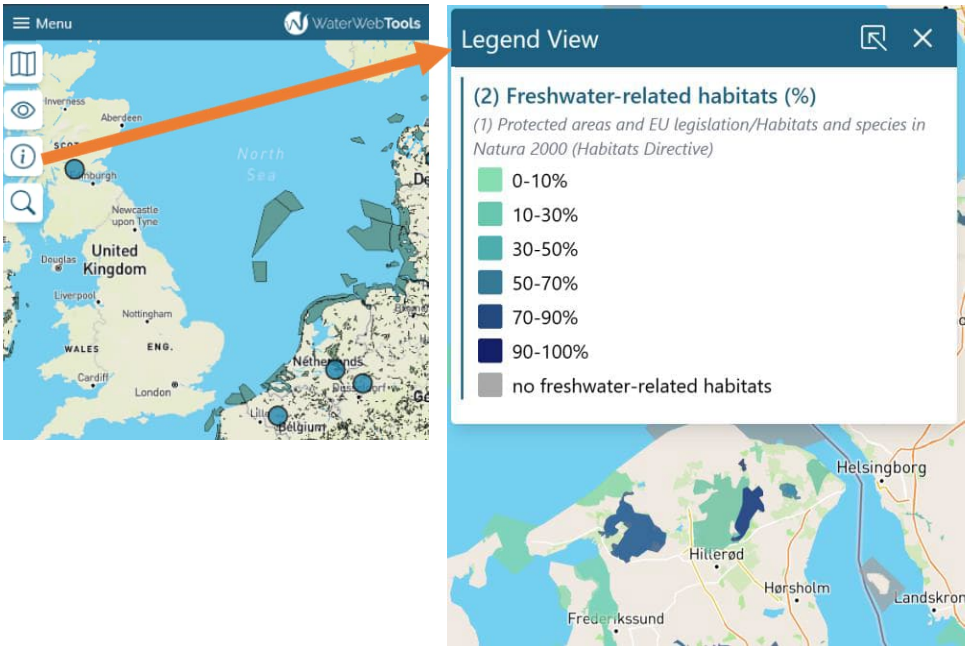

5. To access information on **a specific Natura 2000 site**, click on the site on the map, then go to the Report View to the right and see an overview of protected freshwater-related, terrestrial, marine, and coastal habitats and species.

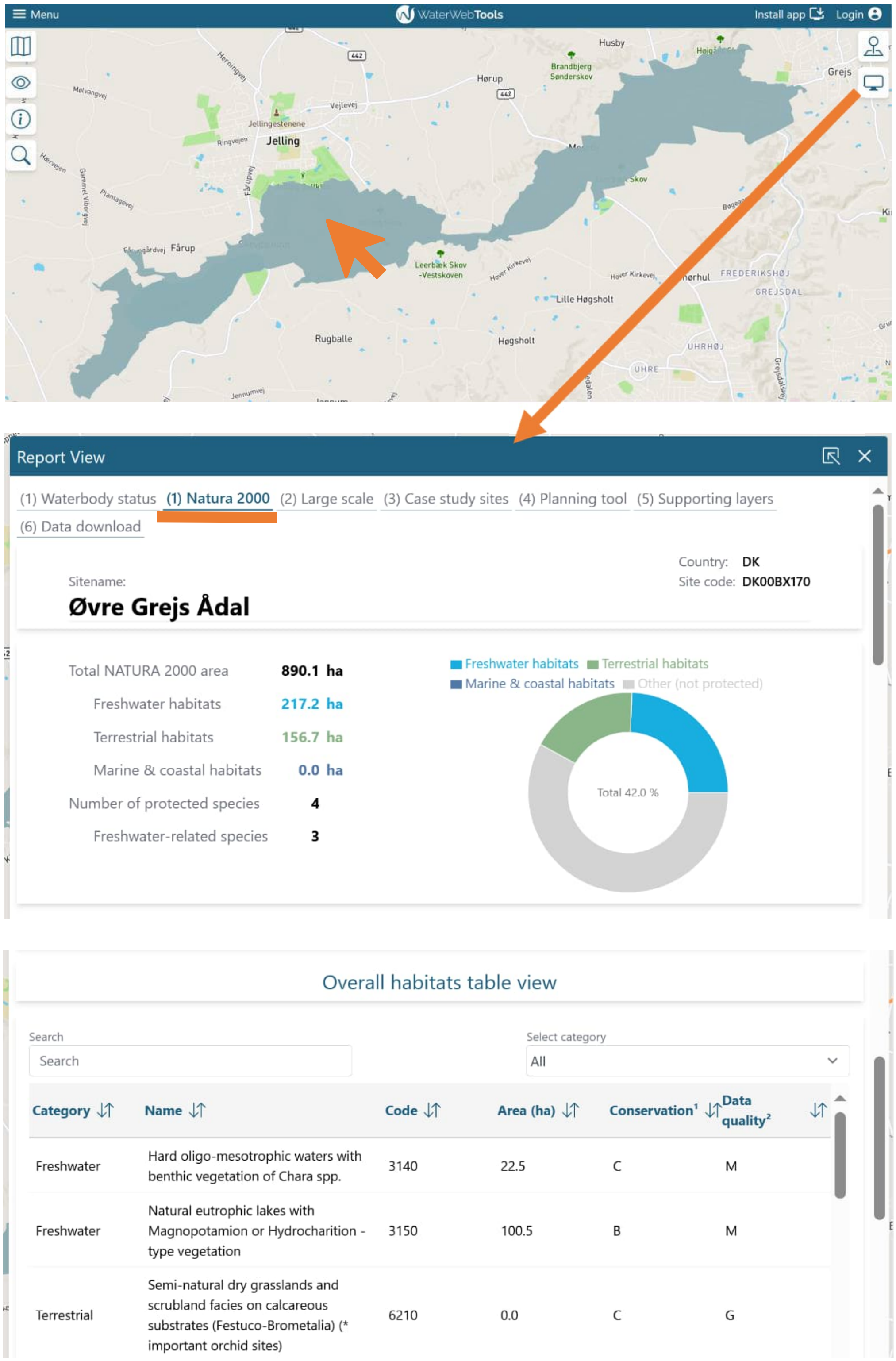

Info about the protected freshwater-related habitats

Area of the habitats	217.2 ha
Number of habitats	10
Standing water habitats	123.0 ha
Running water habitats	0.2 ha
Peatland habitats	23.5 ha
Forest habitats	70.2 ha
Other habitats	0.3 ha

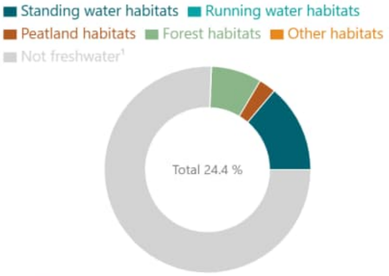

(1): Protected terrestrial, marine and coastal habitats, non-protected habitats, artificial surfaces (e.g., roads, buildinds) etc.

Protected freshwater-related habitats

Search

Search

Select freshwater category

All

Freshwater category	Name	Code	Area	Conservation[1]	Data quality[2]	Link
Standing water	Hard oligo-mesotrophic waters with benthic vegetation of Chara spp.	3140	22.5	C	M	Read more
Standing water	Natural eutrophic lakes with Magnopotamion or Hydrocharition □ type vegetation	3150	100.5	B	M	Read more
Running water	Water courses of plain to montane levels with the Ranunculion fluitantis and Callitricho-Batrachion vegetation	3260	0.2	B	P	Read more

Protected species

Search

Search

Query

All

Group	Scientific name	Legislation[1]	Freshwater[2]	Conservation[3]	Data quality[4]
Fish	Lampetra planeri	HD-II	Yes	C	DD
Mammals	Lutra lutra	HD-II&IV	Yes	C	DD
Invertebrates	Vertigo angustior	HD-II	No	B	DD
Amphibians	Triturus cristatus	HD-II&IV	Yes	C	DD

6. To **search and extract data** write the search terms of interest using the search terms given below the search string. There are many options that can be combined in the search for specific habitats or species. A search could be e.g., member_state=DK, habitat_code=3260 to get all sites with this specific habitat type designated within the member state Denmark or it could be e.g., member_state=DK+PL, scientific_name=Luronium natans to get all sites with this species designated within the network in Denmark and Poland. When searching for features the search terms for habitats are the same as those listed in Annex 1, and for species as those listed in Annexes II and IV and BD Annex I. The code for member state follows the ISO 3166-1 alpha-2 country codes.

   To extract the data, go to the Report View, click download and choose the files and format for the data.

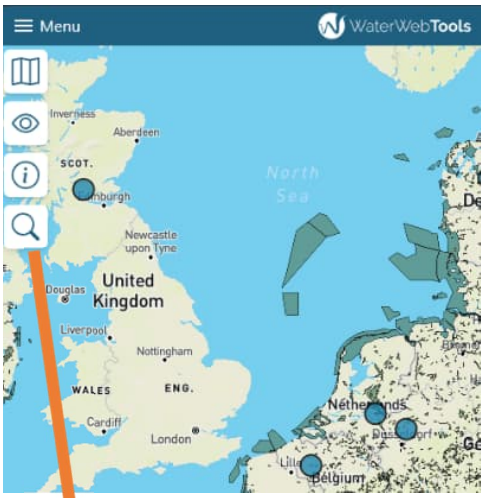

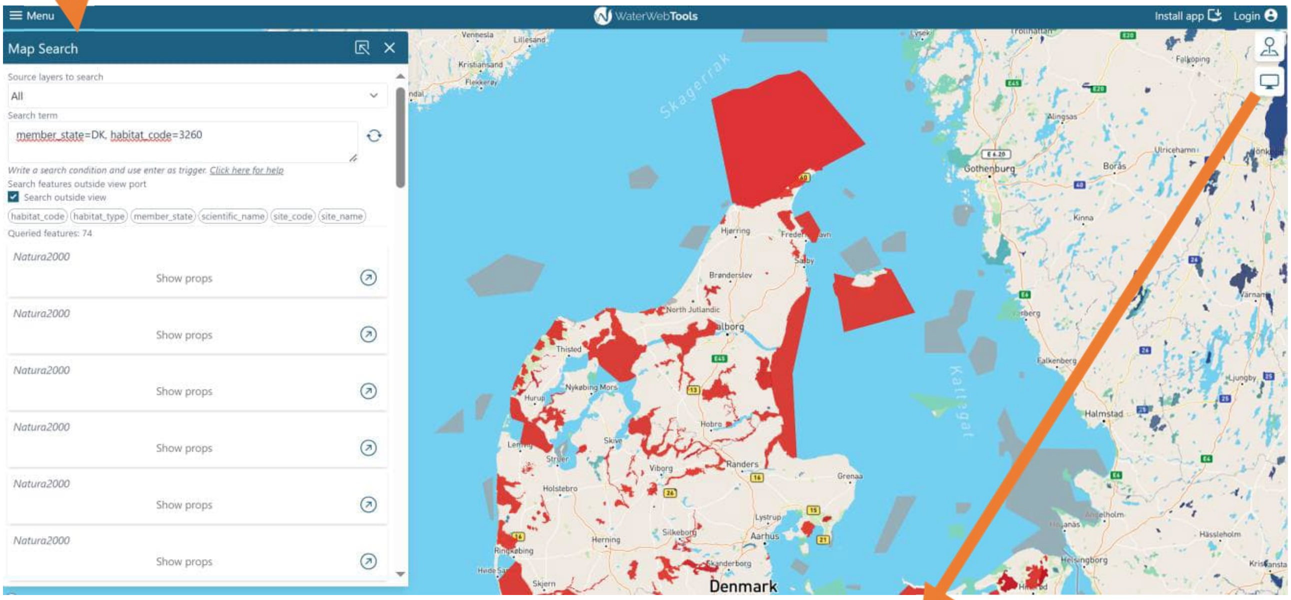

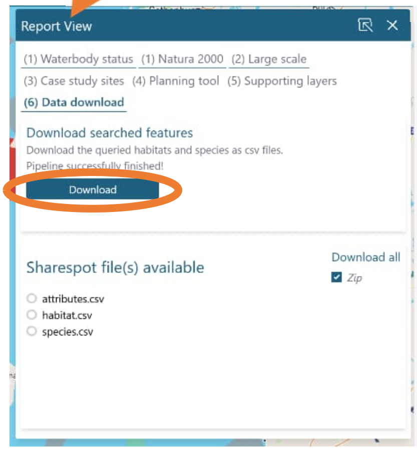

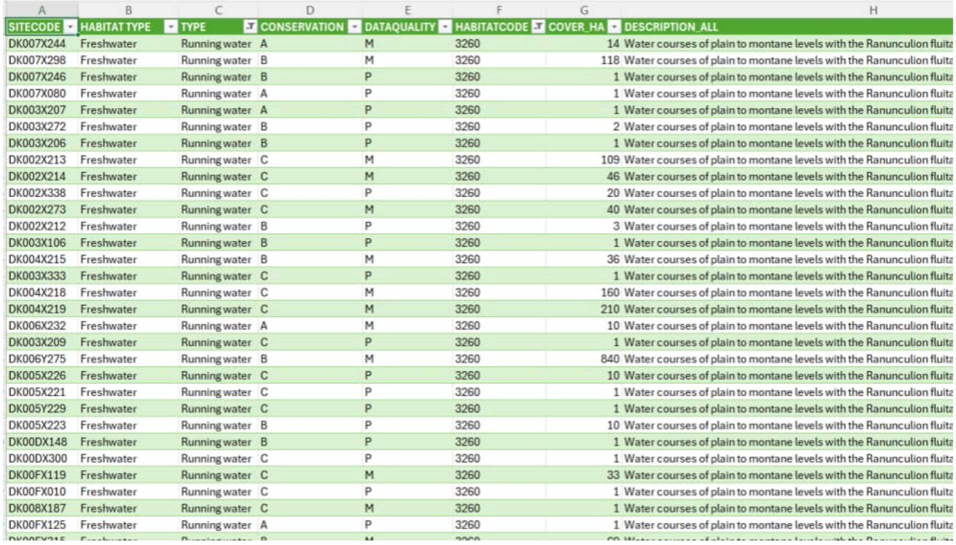

SITECODE	HABITAT TYPE	TYPE	CONSERVATION	DATAQUALITY	HABITATCODE	COVER_HA	DESCRIPTION_ALL
DK007X244	Freshwater	Running water	A	M	3260	14	Water courses of plain to montane levels with the Ranunculion fluita
DK007X298	Freshwater	Running water	B	M	3260	118	Water courses of plain to montane levels with the Ranunculion fluita
DK007X246	Freshwater	Running water	B	P	3260	1	Water courses of plain to montane levels with the Ranunculion fluita
DK007X080	Freshwater	Running water	A	P	3260	1	Water courses of plain to montane levels with the Ranunculion fluita
DK003X207	Freshwater	Running water	A	P	3260	1	Water courses of plain to montane levels with the Ranunculion fluita
DK003X272	Freshwater	Running water	B	P	3260	2	Water courses of plain to montane levels with the Ranunculion fluita
DK003X206	Freshwater	Running water	B	P	3260	1	Water courses of plain to montane levels with the Ranunculion fluita
DK002X213	Freshwater	Running water	C	M	3260	109	Water courses of plain to montane levels with the Ranunculion fluita
DK002X214	Freshwater	Running water	C	M	3260	46	Water courses of plain to montane levels with the Ranunculion fluita
DK002X338	Freshwater	Running water	C	P	3260	20	Water courses of plain to montane levels with the Ranunculion fluita
DK002X273	Freshwater	Running water	C	M	3260	40	Water courses of plain to montane levels with the Ranunculion fluita
DK002X212	Freshwater	Running water	B	P	3260	3	Water courses of plain to montane levels with the Ranunculion fluita
DK003X106	Freshwater	Running water	B	P	3260	1	Water courses of plain to montane levels with the Ranunculion fluita
DK004X215	Freshwater	Running water	B	M	3260	36	Water courses of plain to montane levels with the Ranunculion fluita
DK003X333	Freshwater	Running water	C	P	3260	1	Water courses of plain to montane levels with the Ranunculion fluita
DK004X218	Freshwater	Running water	C	M	3260	160	Water courses of plain to montane levels with the Ranunculion fluita
DK004X219	Freshwater	Running water	C	M	3260	210	Water courses of plain to montane levels with the Ranunculion fluita
DK006X232	Freshwater	Running water	A	M	3260	10	Water courses of plain to montane levels with the Ranunculion fluita
DK003X209	Freshwater	Running water	C	P	3260	1	Water courses of plain to montane levels with the Ranunculion fluita
DK006Y275	Freshwater	Running water	B	M	3260	840	Water courses of plain to montane levels with the Ranunculion fluita
DK005X226	Freshwater	Running water	C	P	3260	10	Water courses of plain to montane levels with the Ranunculion fluita
DK005X221	Freshwater	Running water	C	P	3260	1	Water courses of plain to montane levels with the Ranunculion fluita
DK005Y229	Freshwater	Running water	C	P	3260	1	Water courses of plain to montane levels with the Ranunculion fluita
DK005X223	Freshwater	Running water	B	P	3260	10	Water courses of plain to montane levels with the Ranunculion fluita
DK00DX148	Freshwater	Running water	B	P	3260	1	Water courses of plain to montane levels with the Ranunculion fluita
DK00DX300	Freshwater	Running water	C	P	3260	1	Water courses of plain to montane levels with the Ranunculion fluita
DK00FX119	Freshwater	Running water	C	M	3260	33	Water courses of plain to montane levels with the Ranunculion fluita
DK00FX010	Freshwater	Running water	C	P	3260	1	Water courses of plain to montane levels with the Ranunculion fluita
DK008X187	Freshwater	Running water	C	M	3260	1	Water courses of plain to montane levels with the Ranunculion fluita
DK00FX125	Freshwater	Running water	A	P	3260	1	Water courses of plain to montane levels with the Ranunculion fluita
[illegible]	[illegible]	[illegible]	[illegible]	[illegible]	[illegible]	[illegible]	[illegible]

References

EC (2024). Regulation (EU) 2024/1991 of the European Parliament and of the Council of 24 June 2024 on nature restoration and amending Regulation (EU) 2022/869. Official Journal of the European Union (29.7.2024), 1-93.

EC (2022). Natura 2000 (vector) - version 2021. Retrieved from https://sdi.eea.europa.eu/catalogue/srv/api/records/8c89654c-636a-485f-90b4-3778ea4b7fa3

EC (2009). Directive 2009/147/EC of the European Parliament and of the Council of 30 November 2009 on the conservation of wild birds. Official Journal of the European Union (26.1.2010), 1-19.

EC (2000). Directive 2000/60/EC of the European Parliament and of the Council of 23 October 2000 establishing a framework for Community action in the field of water policy. Official Journal of the European Union (22.12.2000), 1-72.

EC (1992). Council Directive 92/43/EEC of 21 May 1992 on the conservation of natural habitats and of wild fauna and flora. Official Journal of the European Union (22.7.92), 1-50.

EEA (2024). European Red List of Habitats - enhanced by EEA. Retrieved from https://www.eea.europa.eu/en/datahub/datahubitem-view/de2276d8-e295-4cd7-89c9-88812065db87?activeAccordion=

Merlin Webtool (2025). Retrieved from https://www.wwt-platform.com/bgis/d193c35e-9d75-4aea-927e-b220e81fbdfc
